# Seasonal dynamics of bacterial communities in mangrove sediments of Shupaisha island, Zhejiang Province, China

**DOI:** 10.3389/fmicb.2025.1526730

**Published:** 2025-02-19

**Authors:** Maoqiu He, Shoudian Jiang, Xiangfu Li, Liqin Yao, Bonian Shui

**Affiliations:** ^1^Zhejiang Ocean University, Fisheries College, Zhoushan, China; ^2^Fujian Key Laboratory of Marine Carbon Sequestration, Xiamen University, Xiamen, China; ^3^State Key Laboratory of Tropical Oceanography, South China Sea Institute of Oceanology, Chinese Academy of Sciences, Guangzhou, China

**Keywords:** mangrove, Shupaisha island, sediment, bacterial community, seasonal dynamics

## Abstract

Mangrove forests, found primarily in tropical and subtropical coastal regions, support diverse microbial communities that are crucial for nutrient cycling and energy flow and then sustain mangrove ecosystem integrity. This study investigated the seasonal dynamics of bacterial communities in mangrove sediments of Shupaisha island (Zhejiang Province, China) through amplifying and high-throughput sequencing bacterial 16S rRNA gene fragments. Proteobacteria (23.59–44.40%), Actinobacteria (4.92–19.01%), and Bacteroidetes (4.31–22.79%) dominated the bacterial phyla in Shupaisha mangrove sediments with the highest diversity indices in winter. Actinobacteria were more abundant during winter (13.27%) and spring (14.36%), while Bacteroidetes abundance was highest in summer, significantly correlating with temperature. Significant differences in bacterial community composition were observed between winter and summer, while spring and autumn exhibited similar distribution, indicating a transitional pattern in bacterial community dynamics, with temperature and sand content being the most influential factors. This study enhances our understanding of the seasonal characteristics of bacterial communities in the mangrove ecosystems, potentially providing valuable insights into monitoring and assessing the health and stability of mangrove ecosystems in Zhejiang Province.

## 1 Introduction

Mangrove forests are highly productive tropical and subtropical coastal ecosystems characterized by dense stands of salt-tolerant trees and shrubs that thrive in intertidal zones (Feller et al., 2010; Donato et al., 2011; Zhu and Yan, 2022). These forests provide crucial ecological services, including windbreak and wave attenuation, sediment accretion, shoreline stabilization, and seawater purification, earning them the title of “coastal guardians” and highlighting their global ecological, environmental, and economic value (Kathiresan and Bingham, 2001; Lee et al., 2014). Their intricate root systems effectively trap terrestrial sediment, creating a nutrient-rich intertidal habitat supporting high biodiversity, particularly among microorganisms (Srikanth et al., 2016; Bahram et al., 2018; Meng et al., 2022).

Mangrove ecosystem are characterized by a high abundance of bacteria and fungi, comprising 91% of the microbial biomass, with algae and protozoa accounting for 7 and 2%, respectively (Thatoi et al., 2013). Microorganisms play critical roles in organic matter degradation, pollution purification and carbon storage, thus promoting plant growth through the production of phytohormone and siderophore and driving biogeochemical cycles (Holguin et al., 2001; Atwood et al., 2017; Gong et al., 2019; Berlinches de Gea et al., 2023). This important has spurred increasing researches into the value of mangrove microorganisms, contributing to greater efforts in mangrove conservation and restoration. For example, Zhuang et al. found that bacteria enriched in mangrove rhizospheres exhibit significantly higher degradation rates (2–3 times) than those in similar non-mangrove sediment (TieCheng et al., 2000). Abundant bacteria capable of degrading polycyclic aromatic hydrocarbons have been found in mangrove of Jiulong River Estuary (Tian et al., 2008), while *Bacillus* and *Pandoraea* from the Zhanjiang Mangrove Reserve have demonstrated effective degradation of polychlorinated biphenyls (Song et al., 2023). Additionally, dos Santos et al. (2011) and Wu et al. (2016) have explored the composition and functional diversity of mangrove microbial communities, highlighting their importance for maintaining mangrove forests ecological functions. Furthermore, due to the mangroves’ susceptibility to anthropogenic influence, changes in microbial communities can serve as valuable indicators for assessing the pollution levels (Fernandes et al., 2022).

Despite their crucial role in regulating mangrove ecosystems, mangrove microbial communities have received relatively limited attention compared to those in other marine and terrestrial environments (Thompson et al., 2017; Bahram et al., 2018; Thomson et al., 2022). Notably, understanding the temporal and spatial dynamics of microbial communities, and their influencing factors remains a significant challenge. Ho et al. suggested that seasonality may exert a greater influence on microbial communities than forest age (Ho et al., 2024). Wang et al. (2021) demonstrated that total carbon, nitrite, and salinity are important environmental factors that determine the metabolic functional diversity of bacterial communities. These findings underscore the need for further research to unravel the intricate dynamics of microbial communities in mangrove ecosystems (Fierer and Jackson, 2006; Habekost et al., 2008; Lauber et al., 2013; Voříšková et al., 2014; Lan et al., 2018; Zhang et al., 2018).

Shupaisha mangrove, a key protected mangrove wetland in Zhejiang Province, covers approximately 1191.04 hectares (Feng et al., 2024). The region has a mid-subtropical monsoon climate with an average annual temperature of 18°C and rainfall of 1750 mm (Qin et al., 2022). Following the successful restoration of mangroves on Shupaisha Island, through the artificial cultivation of *Kandelia obovata*, the island has become a significant conservation wetland at the northern edge of mangrove forests in China (Wu et al., 2022). However, research in this region has primarily focused on the benthic fauna (Hu et al., 2018), with little attention paid to mangrove microbial communities. Furthermore, its location in the central estuarine area, inaccessible by land and thus minimizing human disturbance, makes it an ideal site for studying the complex dynamics of mangrove microbial communities.

This study aims to investigate the seasonal dynamics of bacterial community structure in Shupaisha mangrove sediment and identify key environmental regulatory factors. Using high-throughput sequencing of bacterial 16S rRNA gene fragments, we have analyzed the bacterial community composition across seasons. Concurrently, various environmental factors, including temperature, salinity, pH, total carbon, and total nitrogen were measured and statistically analyzed with bacterial communities. These findings will contribute to a deeper understanding of mangrove ecosystems, providing a theoretical basis for promoting sustainable development and ecological restoration of mangrove forests.

## 2 Materials and methods

### 2.1 Sampling design and collection

**Figure 1:**
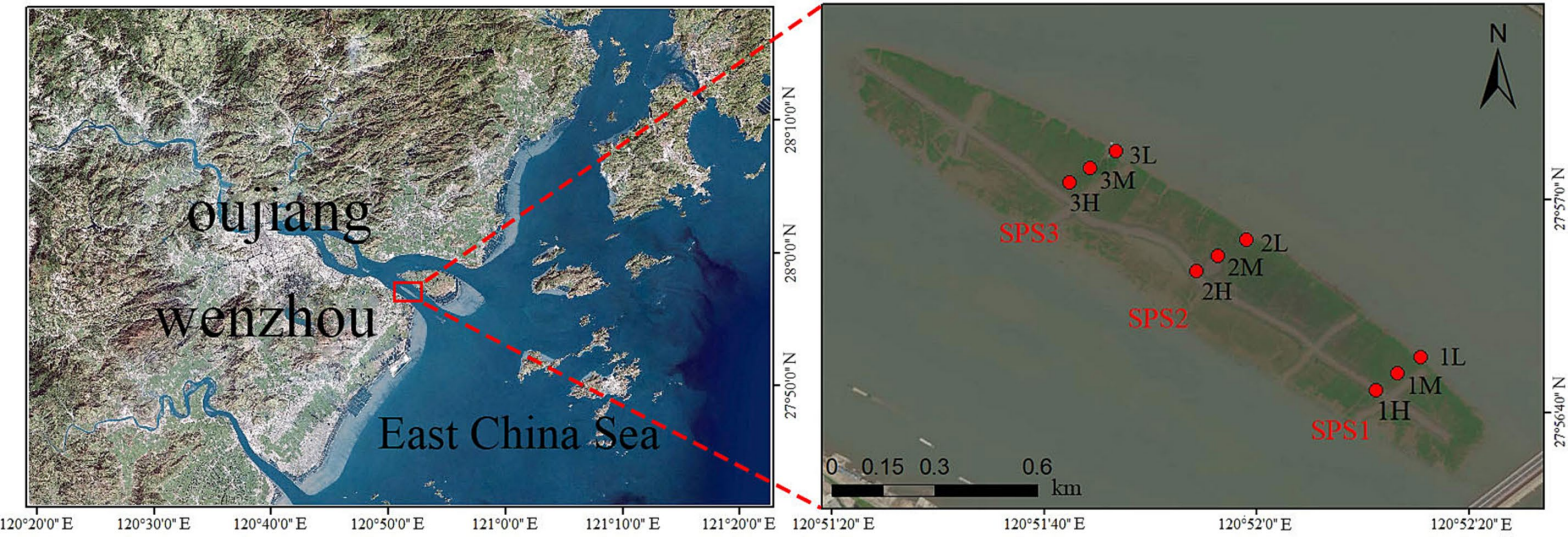
Satellite map of sampling sites in mangrove forest of Shupaisha island.

Sediment samples were collected from three transects (SPS1, SPS2, and SPS3) within the intertidal zone of Shupaisha Island mangrove forest during four seasons: December 2021 (winter), May 2022 (spring), August 2022 (summer), and November 2022 (autumn) (Figure 1). Within each transect, three sampling sites were established representing the high, mid, and low tidal zones. Sampling sites were carefully selected to avoid obvious anthropogenic disturbances. The top 1 cm of sediment was removed, and samples were collected using a 30 mL sterile syringe with the head removed. Collected sediment was transferred to 50 mL sterile centrifuge tubes and stored temporarily at −20°C. Samples were then transported to the laboratory as soon as possible and stored at −80°C until further processing.

### 2.2 Environmental parameter measurement of mangrove sediments

Temperature, salinity, and pH were measured *in situ* using a portable multi-parameter water quality analyzer (YSI ProPlus, USA). Sediment particles size analysis was performed using a laser particle size analyzer (Bettersize2600, Dandong) following a standard protocol. This involved mixing 2 g of sediment with 3 mL of 0.5 mol/L sodium hexametaphosphate solution for 2 min, allowing the mixture to stand for 8 h, shaking it again, and then adding several drops to the analyzer. Total carbon content (TC) and total nitrogen content (TN) were determined using an elemental analyzer (Thermo Fisher Scientific FLASH2000, Europe) according to the manufacturer’s instructions.

### 2.3 DNA extraction, PCR amplification, and sequencing

Sediment samples were processed for microbial DNA extraction using the DNeasy PowerSoil Pro kit (Qiagen), following the manufacturer’s instructions. DNA concentration was subsequently quantified using a NanoDropTM One micro-volume spectrophotometer. With sediment DNA as templets, the V3-V4 region of the bacterial 16S rRNA gene was amplified using primers 341F (5′-CCTAYGGGRBGCASCAG-3′) and 806R (5′-GGACTACNNGGG TATCTAAT-3′) (Wu et al., 2016). Each PCR reaction was performed in a 25 μL (final volume) mixture containing 2 × KAPA HiFi HotStart ReadyMix (12.5 μL), 5 μL of Primer 343F (final concentration of 1 μM) and Primer 798R (final concentration of 1 μM), and 2.5 μL template DNA (final concentration of 5 ng μL^−1^). The PCR cycling conditions were as follows: starting with a 3 min initial denaturation at 95°C, followed by 25 cycles of denaturing at 95°C for 30 s, annealing at 60°C for 30 s, extension at 72°C for 30 s, respectively, and a final extension at 72°C for 10 min. PCR products were examined using 1% agarose gel electrophoresis, purified using AMPure XP beads and quantified using the Qubit dsDNA HS assay kit. Amplified fragments were subsequently prepared into libraries according to the standard protocol of the NEBNext UltraTM II DNA Library Prep Kit for Illumina (New England Biolabs, USA). The constructed amplicon libraries were sequenced on the Illumina Nova 6,000 platform using PE250 sequencing (Guangdong Magigene Biotechnology Co., Ltd. Guangzhou).

### 2.4 Processing of sequence data

Quality control, filtering, and paired-end sequence merging of raw sequences were performed using DADA2 in QIIME2, with parameters set as follows: -p-trim-left-*f* = 23, −p-trim-left-r = 26, −p-trunc-len-*f* = 250, and -p-trunc-len-r = 250. This resulted in the generation of amplicon sequence variant (ASV) and a feature table. ASVs were then clustered into operational taxonomic units (OTUs) at a 97% similarity using the search cluster-features-de-novo algorithm (Uroz et al., 2010). Naïve Bayes classifiers for both Silva (version 138.2) and Greengenes2 database were trained using the qiime-feature-classifier algorithm (DeSantis et al., 2006; Quast et al., 2013; McDonald et al., 2024). Bacterial OTUs were subsequently classified using the classify-sklearn command based on these two classifiers. A frequency threshold greater than 2 and a total sequencing depth greater than 10 reads were applied to filter OTUs, resulting in a final OTU table. All sequences obtained from this study have been deposited in the National Center for Biotechnology Information GenBank (NCBI) under accession number PRJNA1133986.

### 2.5 Bacterial diversity and statistical analysis

Richness and Shannon indices were calculated using R software (V4.3.2) (Null et al., 2011) and statistically analyzed using Origin 2021 and SPSS statistical software (V27.0). One-way ANOVA and Kruskal-Wallis tests were conducted to compare environmental parameters and diversity indices of the microbial community across different seasons. The relative abundances of dominant phyla and families were calculated and visualized using stacked bar charts. Heatmaps were generated using R (“pheatap” package) to depict species abundance across samples and calculate correlations between bacterial community components and environmental factors. Redundancy analysis (RDA), principal component analysis (PCA), and analysis of similarities (ANOSIM) were performed using R software. The STAMP software was used to analyze bacterial families exhibiting significant differences between groups. PICRUSt2 software was used to predict the metabolic functions of bacterial communities in mangrove sediment samples based on KEGG database comparisons (Langille et al., 2013).

## 3 Results

### 3.1 Environmental parameters of Shupaisha mangrove sediment

This study measured environmental parameters including temperature, salinity, pH, total carbon content (TC), and total nitrogen content (TN) in sediment samples collected from Shupaisha mangrove during different seasons (Table 1). Temperature exhibited significant seasonal variations, ranging from 13.93 ± 0.52°C in winter to 31.31 ± 3.07°C in summer. The lowest pH was observed in spring (7.26 ± 0.27), while no significant difference was observed between other three seasons. For all season sediments, TC ranged from 12.74 ± 1.33 to 13.00 ± 0.90 mg/g, TN ranged from 1.13 ± 0.15 to 1.17 ± 0.09 mg/g, and TC/TN ratios ranged from 11.03 ± 0.66 to 11.35 ± 1.12. Unexpectedly, no significant seasonal differences were observed in any of these parameters. In terms of sediment particle size, the lowest clay content was found in winter sample (35.17 ± 2.09%), and no significant difference was observed among other three seasons. Additionally, sand content was significantly higher in winter compared to in summer and autumn (*p* < 0.05), while silt content remained stable across all seasons. Based on the soil classification system, the sediment of Shupaisha mangrove was generally classified as loam (Ditzler, 2017). The flood season in the Oujiang River Basin typically extends from April to September due to the sustained impact of warm front and typhoon (Wanying et al., 2023). However, a drought in the summer of 2022 caused a decrease in Oujiang River runoff, resulting in the highest of river runoff in the spring of 2022. It’s well known that greater runoff corresponds to lower salinity in estuarine areas, therefore, salinity was significantly lower in spring (12.77 ± 2.37) and higher in autumn (30.47 ± 4.05).

**Table 1:** Mangrove sediment environmental parameters of different seasons.

Environmental parameters	Winter	Spring	Summer	Autumn
Temp (°C)	13.93 ± 0.52^d^	18.50 ± 0.75^c^	31.31 ± 3.07^b^	19.96 ± 0.52^a^
SAL (ppt)	20.33 ± 2.56^b^	12.77 ± 2.37^c^	21.13 ± 3.15^b^	30.47 ± 4.05^a^
pH	7.79 ± 0.18^ab^	7.26 ± 0.27^c^	7.96 ± 0.50^a^	7.58 ± 0.35^b^
Clay (%)	35.17 ± 2.09^b^	39.66 ± 1.51^a^	38.65 ± 3.27^a^	38.78 ± 2.74^a^
Silt (%)	61.90 ± 3.09^a^	59.45 ± 1.76^a^	60.58 ± 3.35^a^	60.50 ± 3.09^a^
Sand (%)	2.92 ± 3.51^a^	0.97 ± 0.52^ab^	0.77 ± 0.70^b^	0.71 ± 1.08^b^
TC (mg/g)	12.75 ± 1.22^a^	13.00 ± 0.90^a^	12.74 ± 1.33^a^	12.88 ± 3.36^a^
TN (mg/g)	1.16 ± 0.12^a^	1.17 ± 0.09^a^	1.13 ± 0.15^a^	1.14 ± 0.24^a^
TC/TN	11.03 ± 0.66^a^	11.15 ± 0.41^a^	11.35 ± 1.12^a^	11.28 ± 0.77^a^

Different letters in the same row indicated significant differences (*p* < 0.05); Temp: Temperature; SAL: salinity; TC: total carbon; TN: total nitrogen; TC/TN: total carbon to total nitrogen ratio.

### 3.2 Diversity indices of mangrove bacterial communities

A total of 2,527,395 sequences remained from the 36 samples in this study, resulting in 12,188 OTUs based on a 97% similarity. Rarefaction curves indicated that the sequencing depths were adequate to reflect the actual microbial community composition of the sedimentary samples (Supplementary Figures S1, S2). ANOVA analysis revealed significant differences in richness indices among different seasons (*p* < 0.05), with spring (1,111 ± 125) < autumn (1,608 ± 225) < summer (1815 ± 198) < winter (2,198 ± 119) (Table 2). Similarly, Shannon indices were highest in winter (9.065 ± 0.122) and significantly different from spring (8.424 ± 0.176) and summer (8.775 ± 0.270) (*p* < 0.05). However, there were no significant differences in diversity indices between different transects and tidal zones, suggesting that bacterial communities in Shupaisha mangrove did not exhibit spatial heterogeneity within the same season.

**Table 2:** Diversity index of bacterial community in Shupaisha mangrove sediments.

	Richness	Shannon	Simpson	Pielou
Winter	2,198 ± 119^a^	9.065 ± 0.122^a^	0.994 ± 0.000^a^	0.817 ± 0.010^ab^
Spring	1,111 ± 125^d^	8.424 ± 0.176^c^	0.992 ± 0.002^a^	0.833 ± 0.015^a^
Summer	1815 ± 198^b^	8.775 ± 0.270^b^	0.992 ± 0.003^a^	0.811 ± 0.021^b^
Autumn	1,608 ± 225^c^	8.878 ± 0.366^ab^	0.992 ± 0.004^a^	0.834 ± 0.023^a^
High tidal zone	1,670 ± 383	8.824 ± 0.307	0.993 ± 0.002	0.827 ± 0.010
Middle tidal zone	1,690 ± 470	8.890 ± 0.283	0.994 ± 0.001	0.834 ± 0.020
Low tidal zone	1,687 ± 471	8.642 ± 0.389	0.991 ± 0.003	0.811 ± 0.022

Different letters in the same column indicated significant differences between different seasons (*p* < 0.05).

### 3.3 Spatial and temporal characteristics of bacterial community composition

**Figure 2:**
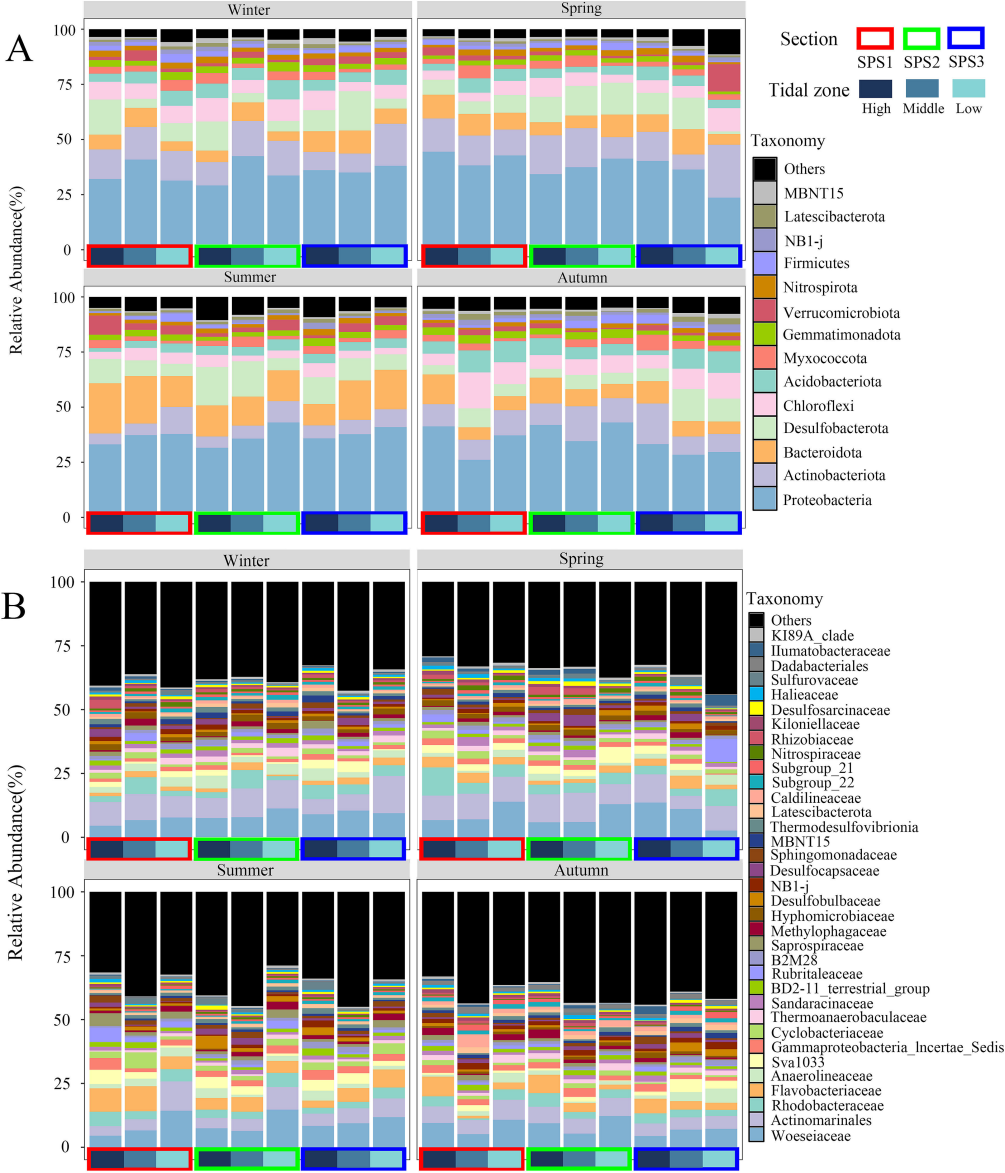
Distribution of abundant bacterial phyla **(A)** and families **(B)** in Shupaisha mangrove sediment during different season.

All OTUs were classified into 64 phyla and 736 families. The distribution of dominant bacterial phyla and families was presented in Figures 2A,B, respectively. Fourteen phyla with relative abundance greater than 1% were identified, accounting for over 90% of the sequences in the corresponding samples. These phyla were ranked from highest to lowest relative abundance: Proteobacteria (23.59–44.40%), Actinobacteria (4.92–19.01%), Bacteroidetes (4.31–22.79%), Desulfobacterota (3.95–17.78%), Chloroflexi (2.61–16.30%), Acidobacteriota (1.70–9.98%), Myxomycota (1.30–7.10%), Gemmatimonadetes (1.29–4.17%), Verrucomicrobia (0.26–12.32%), Nitrospirae (0.73–3.00%), Firmicutes (0.12–4.01%), NB1-j (0.33–2.77%), Latescibacterota (0.32–2.69%), and MBNT15 (0.31–2.70%).

The relative abundance of Actinobacteria was significantly higher in winter (13.27%) and spring (14.36%) compared to summer (7.04%) and autumn (11.32%). In summer, the average relative abundance of Chloroflexi (4.28%) and Acidobacteriota (3.79%) was significantly lower than in the other three seasons, while Bacteroidetes (16.14%) exhibited a contrary tendency. Additionally, the relative abundance of Desulfobacterota in autumn samples (7.15%) was lower than in the other three seasons (Figure 2A). In summer, Proteobacteria and Actinobacteria showed a decreasing trend from the high intertidal zone to the low intertidal zone, while no similar trend was observed during other seasons. Furthermore, in the low intertidal samples from transect SPS3 in spring, the relative abundance of Actinobacteria, Chloroflexi, and Verrucomicrobia was higher than in other samples, indicating a distinct site-specific distribution.

At the family level, the top 35 bacterial families accounted for over 60% of the total sequences (Figure 2B). Dominant families included Woeseiaceae, Actinomarinales, Rhodobacteraceae, Flavobacteriaceae, Anaerolineaceae, Sva1033, and Incertae_Sedis group within the order Proteobacteria, with average relative abundances of 8.59, 7.77, 3.99, 3.74, 2.49, 2.46, and 2.09%, respectively. Similar to the phylum distribution, Actinomarinales exhibited higher relative abundances in winter (9.41%) and spring (9.51%) compared to summer (6.15%) and autumn (6.00%). Flavobacteriaceae (6.04%) and Cyclobacteriaceae (2.80%), both belonging to Bacteroidetes, had higher relative abundance in summer. Notably, Woeseiaceae, belonging to Proteobacteria, was predominant in the low tide zones across various seasons, with the exception of the low tide zone from transect SPS3 during the winter and spring. Regarding tidal zone distribution, during the summer, the relative abundance of Actinomarinales increased from the high tide zone to the low tide zone, whereas that of Sva1033 decreased. However, no similar tendency was observed during the remaining seasons.

**Figure 3:**
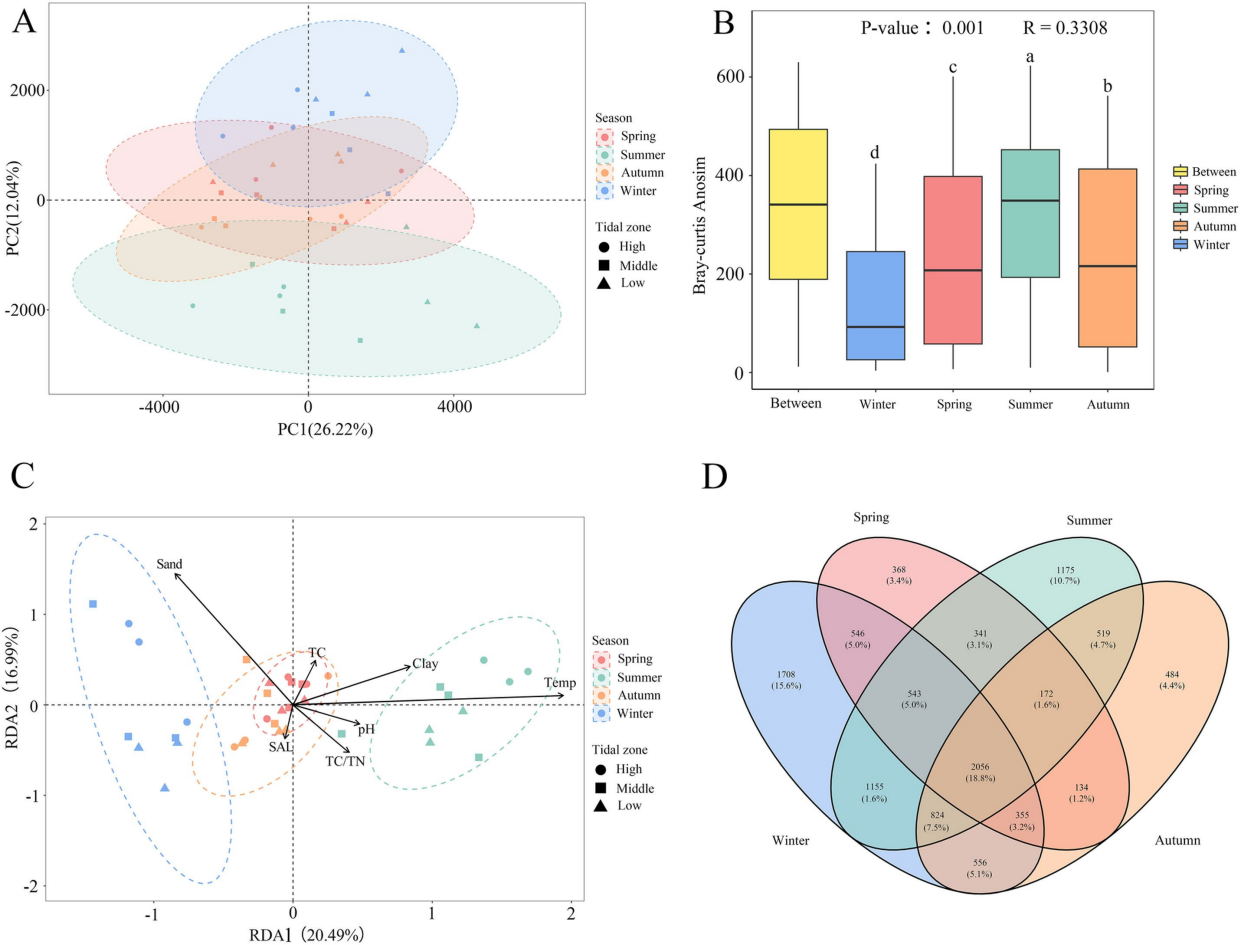
Principal component analysis **(A)**, Anosim analysis **(B)**, RDA analysis **(C)** and Venn diagram of bacterial community structure in different seasons **(D)**. The color represents season; the dot shape represents the tidal zone; different letters indicate significant differences (*p* < 0.05).

Principal component analysis (PCA) results revealed distinct seasonal characteristics of bacterial community structure in mangrove sediment. PC1 accounted for 26.22% of the variation, while PC2 accounted for 12.04% (Figure 3A). Samples from winter and summer were clearly separated into two distinct clusters, indicating significant seasonal differences in bacterial community composition. In contrast, samples from spring and autumn were highly overlapped, suggesting the similarity. Furthermore, samples from different intertidal zones were not clearly separated and were intermingled, indicating no significant differences in bacterial community across different tidal levels (*p* > 0.05). Anosim analysis with season as a factor showed significant differences in bacterial community composition between different seasons (*p* < 0.01, *R* = 0.33). Except for summer when the rank was higher for the “Between” group, the differences in bacterial community composition between groups were greater than within groups in other seasons (Figure 3B).

A Venn diagram illustrated the shared and special OTUs (those present only in a single season) among sediment samples from different season (Figure 3D). There were 2056 OTUs that shared among all seasons and occupied 18.8% of all OTUs, demonstrating a degree of stability in bacterial community through the whole year. The distribution of special OTUs appeared the obvious seasonal characteristic, with the higher number of special OTUs in winter (1708) and summer (1175) compared to autumn (484) and spring (368). This suggests a significant distinctness in the bacterial communities of sediment samples from winter and summer.

**Figure 4:**
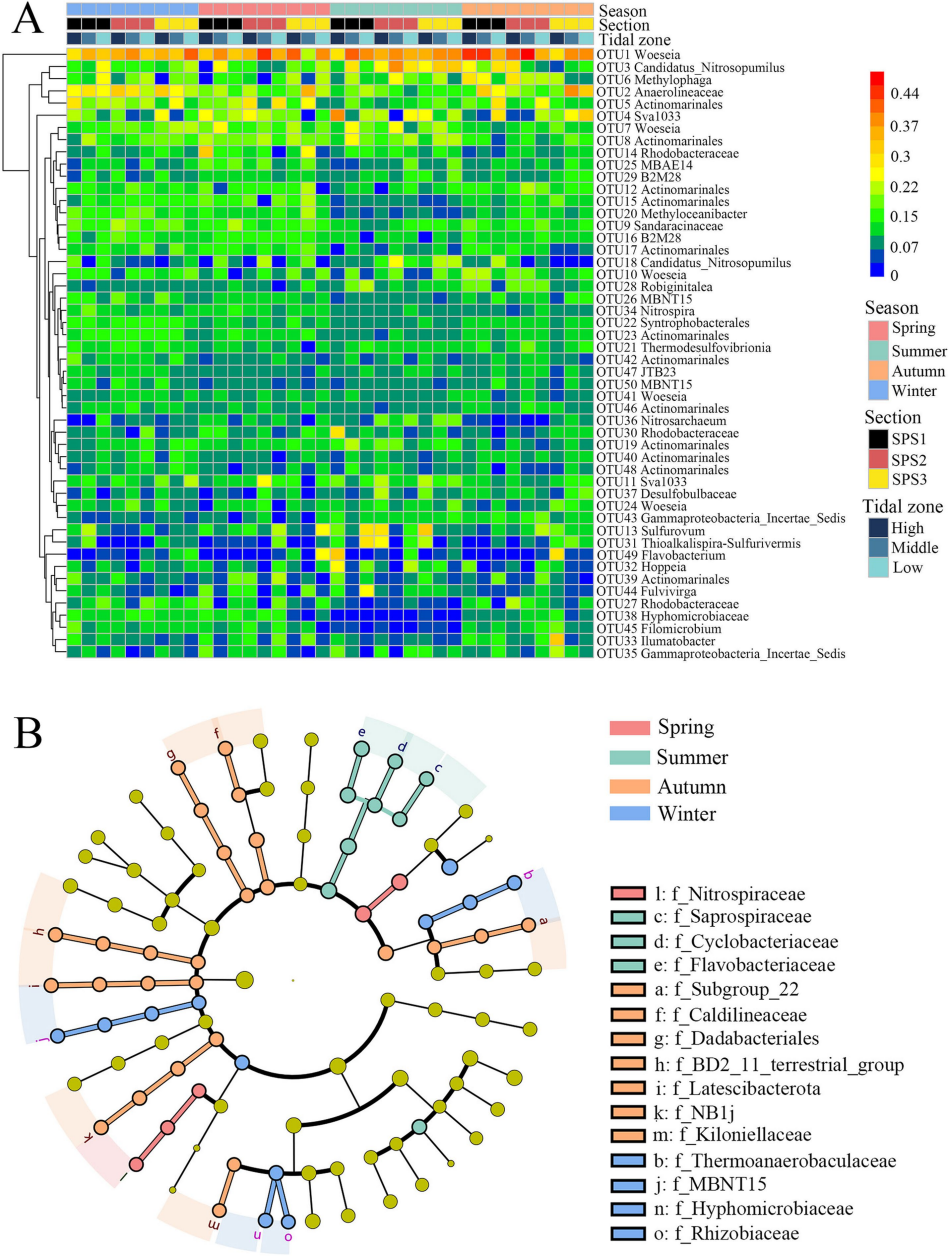
Heatmap of the top 50 OTUs in all sediment samples **(A)** and Lefse analysis of bacterial families with significant difference across four seasons **(B)**.

The top 50 OTUs were classified and analyzed as heatmap (Figure 4A). The original data was square-root transformed before analysis. OTU1 (*Woeseia*) exhibited relatively high abundance in all seasons, particularly in transects SPS1 and SPS2 during autumn. In summer, several OTUs showed distinct differences compared to other seasons. OTU12, OTU15, and OTU17 belonging to Actinomarinales, and OTU20 (*Methyloceanibacter*), OTU9 (Sandaracinaceae), OTU16 (*B2M28*) exhibited significantly lower relative abundance, while OTU24 (*Woeseia*), OTU43 (Incertae_Sedis group within Proteobacteria), OTU13 (*Thiobacillus*) and OTU31 (*Thioalkalispira-Sulfurivermis*) showed the opposite trend with higher relative abundance. Furthermore, OTU27 (Rhodobacteraceae), OTU38 (Saprospiraceae), and OTU45 (*Leifsonia*) clustered together and showed low relative abundance in summer but high relative abundance in winter, spring, and autumn. Notably, OTU49 (*Flavobacterium*) with high relative abundance was only occasionally observed in the low intertidal zone of SPS3 in spring, the high intertidal zone of SPS1 in summer, and the high intertidal zone of SPS3 in autumn.

Pairwise comparisons of the relative abundances of the top 35 bacterial families across the four seasons revealed significant differences (Supplementary Figure S3). Twelve families showed significant differences between summer and the other three season. Compared to summer, Flavobacteriaceae and Incertae_Sedis group were significantly less abundant in winter, while Cyclobacteriaceae and Saprospiraceae were less abundant in autumn. Conversely, Hyphomicrobiaceae, Caldilineacea, Nitrospiraceae, Actinomarinales, Rhizobiaceae, and Thermoanaerobaculaceae were more abundant in spring. Furthermore, Linear discriminant analysis effect size (LefSe) analysis further identified bacterial families with significant seasonal differences (Figure 4B). Thermoanaerobaculaceae, MBNT15, Hyphomicrobiaceae and Rhizobiaceae were enriched in winter; Nitrospiraceae in spring; Saprospiraceae, Cyclobacteriaceae, Flavobacteriaceae in summer, and Subgroup 22 (Acidobacteriota), Caldilineaceae, BD2_11, Latescibacterota, NB1_j and Kiloniellaceae in autumn.

### 3.4 Effects of environmental factors on mangrove bacterial community

Redundancy analysis (RDA, Figure 3C) was conducted after removing TN and silt content due to collinearity. The first RDA axis explained 20.49% of the variation, while the second axis explained 16.99%, accounting for a total of 37.48% of the variation. The first axis was positively correlated with temperature, clay content, pH, TC, and TC/TN ratio, but negatively correlated with sand content and salinity. The second axis was positively correlated with sand content, temperature, clay content, and TC, but negatively correlated with salinity, pH, and TC/TN ratio. Among these factors, temperature and sand content exerted the greater influences on bacterial community composition. Similar to the PCA, winter and summer samples were distributed in different regions, indicating significant differences in bacterial community composition between these two seasons. Furthermore, spring and autumn samples were highly overlapped, suggesting a high degree of similarity.

**Figure 5:**
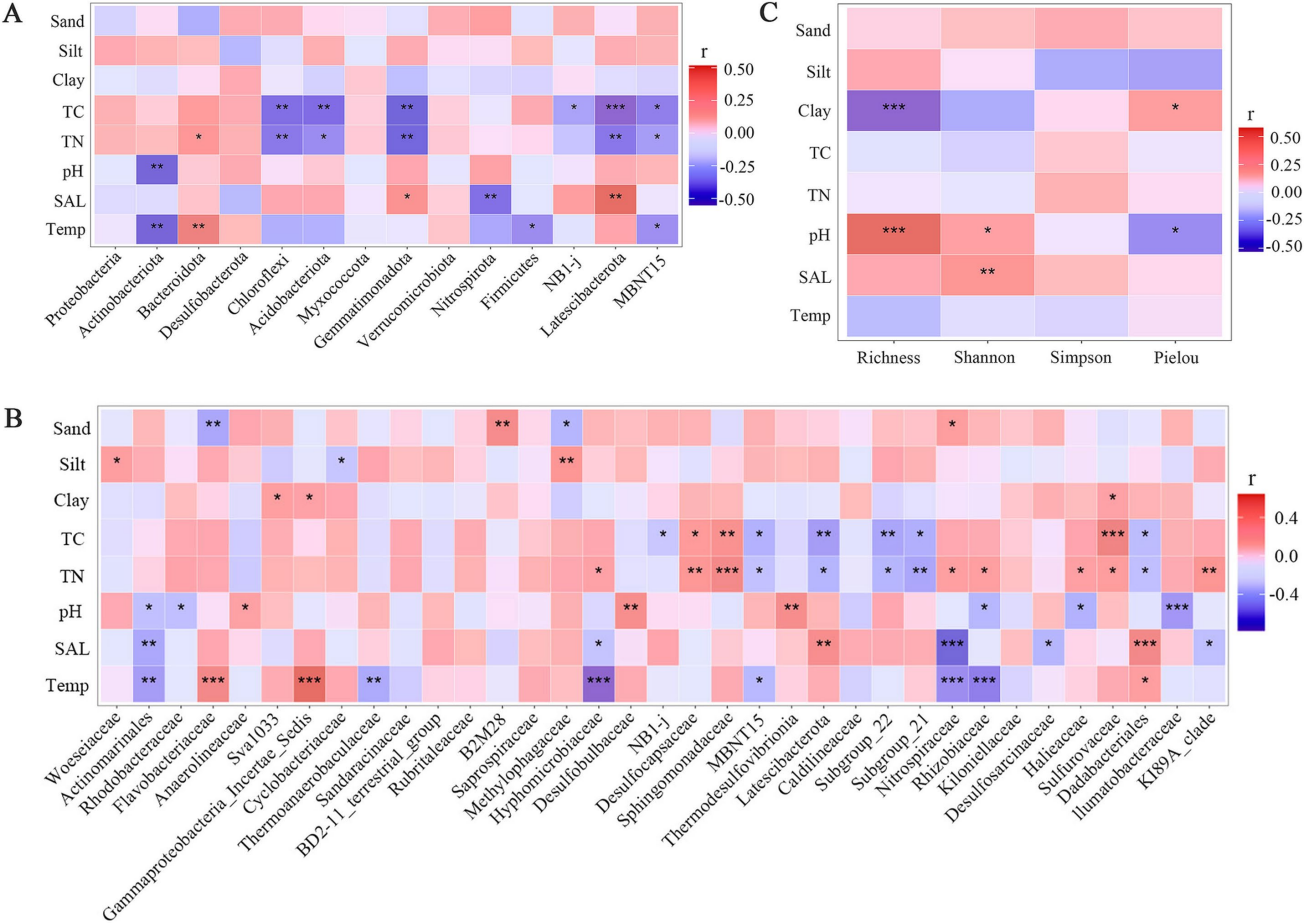
Heatmap of the correlation between bacteria diversity indices **(A)**, abundant bacterial phyla **(B)** and bacterial families **(C)** and environmental factors, respectively. Temp: Temperature; SAL: salinity; TN: Total Nitrogen; TC: Total Carbon; *p < 0.05; **p < 0.01; ***p < 0.001.

Correlation analysis revealed numerous significant relationships between bacterial community diversity indices, the relative abundances of abundant phyla and top 35 families, and environmental factors, respectively (Figure 5). A significant positive was observed between richness index and pH (*R* = 0.51, *p* < 0.001), while a negative correlation was found between richness and clay content (*R* = −0.52, *p* < 0.001). Additionally, Shannon index was positively correlated with pH (*R* = 0.37, *p* < 0.05) and salinity (*R* = 0.51, *p* < 0.01, Figure 5C).

Five phyla, including Chloroflexi, Acidobacteriota, Gemmatimonadota, Latescibacterota and MBNT15, exhibited negative correlations with both TC and TN, suggesting the regulation of substrate concentration on bacterial communities in mangrove sediment (Figure 5A). Furthermore, the relative abundances of Actinobacteria, Firmicutes, and MBNT15 were significantly negatively correlated with temperature, while Bacteroidetes showed positive correlation, consistent with the distribution pattern of bacterial phylum across different seasons (Figure 2A). Only one phylum showed a significant correlation with pH, three phyla exhibited significant correlation with salinity.

The richer correlations were observed at the family level (Figure 5B). Similar to bacterial phyla, TN, TC and temperature existed a large number of significant correlations between top 35 families, with 13, 9 and 9 correlations, respectively. However, several families showed the significant correlations between particle size parameters (sand, silt and clay content), a pattern not observed at the phylum level. For instance, Flavobacteriaceae and Methylophaga were negatively correlated with sand content, while B2M28 and Nitrospiraceae was positive correlated. Additionally, eight and seven families showed the significant correlations with pH and salinity, respectively. Notedly, at both the phylum and family level, several bacterial groups with the higher relative abundance were not significantly correlated with TC and TN.

### 3.5 Metabolic function of bacterial communities in mangrove sediments

PICRUSt2 software was used for the comparative analysis of all OTUs against the Kyoto Encyclopedia of Genes and Genomes (KEGG) database. This analysis yielded 45 function categorizations of the bacterial community, grouped into six groups: Metabolism, Genetic Information Processing, Environmental Information Processing, Human Diseases, Cellular Processes, and Organismal Systems (Supplementary Figure S3). The majority of functional categorizations belonged to Metabolism, Genetic Information Processing and Environmental Information Processing, with “Global and overview maps” being the most prevalent across all samples. Furthermore, there are also significant seasonal differences, with winter and summer abundances being higher than those observed in the other two seasons. In winter, the abundance of metabolic functions was significantly greater than in the other seasons, particularly for “Carbohydrate metabolism,” “Amino acid metabolism,” “Biosynthesis of other secondary metabolites,” “Xenobiotics biodegradation and metabolites,” and “Metabolism of other amino acids.”

## 4 Discussion

Mangrove microbial communities play crucial roles in various ecological functions, such as pollutant degradation and biogeochemical cycling (Bag et al., 2022; Zhang et al., 2023; Qian et al., 2024; Yan et al., 2024), making microbial composition and biological processes valuable indicators of mangrove preservation status (Ma et al., 2021; de Carvalho et al., 2024; Siddique et al., 2024). This study aimed to investigate the temporal and spatial characteristics of sediment microbial communities in Shupaisha mangrove, enhancing our understanding of artificial mangrove ecosystems.

### 4.1 Diverse bacteria in Shupaisha mangrove sediment

A total 64 phyla and 736 families were identified in Shupaisha mangrove sediment, with Shannon indices ranging 8.06 from 9.25 (mean value: 8.78 ± 0.34). These values were compared to those observed in Daya Bay (7.9–9.9) and Golden Bay (8.0–9.0) (Zhu et al., 2018), exceeding those found in Zhangjiang River (5.16–5.23) (Gao et al., 2019), Shenzhen (6.45–7.81) (Ma et al., 2020) and Bamen Bay (5.10–5.85) (Liu et al., 2019), but lower than those in Beibu Gulf (9.44–10.46) (Gong et al., 2019). These variations in bacterial diversity could be attributed to differing levels of artificial pollution. Despite Shenzhen mangrove is adjacent to the Daya Bay, the diversity index of former was lower, primarily due to the inhibitory effects of heavy metal pollution on bacterial diversity (Ma et al., 2020; Zhang et al., 2022). Similarly, Shupaisha mangrove, located in the central part of Oujiang River with no land access, experiences minimal artificial contaminant, potentially contributing to its relatively high bacterial diversity.

Proteobacteria, Bacteroidetes, Chloroflexi, and Acidobacteria were identified as the dominant phyla in this study, accounting for 36.29, 9.97, 6.72, and 5.31% of total abundance, respectively (Figure 3A).This finding aligns with previous surveys along the southeastern coast of China (Ma et al., 2020). Similar dominance of these phyla has been observed in mangrove of Guangxi and Shenzhen (Liao et al., 2020), as well as in the mangrove wetlands of the Beibu Gulf, where they collectively accounted for approximately 80% of the total bacterial abundance (Gong et al., 2019).

Proteobacteria, the most dominant bacterial phylum, are globally distributed in mangrove sediment and are regarded as key roles in carbon, nitrogen, and phosphorus cycling, as well as pollution remediation (Ma et al., 2020; Zhang et al., 2023). Chloroflexi, facultative anaerobic bacteria, are capable of utilizing recalcitrant organic matters, leading to their prevalence in tidal flat sediments below 2 m and serving as an indicator of environmental pollution (Wilms et al., 2006; Zhu et al., 2018). Acidobacteria are involved in iron cycling and the metabolism of single-carbon compounds (Guang-Hua et al., 2016), while Bacteroidetes are often associated with phosphorus cycling (Liu et al., 2020). In Shupaisha mangrove, the detritus from artificially introduced *Kandelia obovata* primarily consists of cellulose, lignin, and other natural organic compounds (Chen et al., 2023). This substantial accumulation of organic detritus provides an optimal environment for microbial communities. Correspondingly, the abundance of bacteria greatly contributes to the degradation of organic matter and nutrients transformation, supporting the high productivity of mangrove ecosystems (Donato et al., 2011; Becker et al., 2020).

### 4.2 Seasonal dynamics of mangrove bacterial community

In this study, winter and summer samples exhibited significantly higher alpha diversity indices compared to spring and autumn. Specifically, mean richness index of winter samples (2,197 ± 118) was approximately twice that of spring samples (1,111 ± 125) (Table 2), indicating a clear seasonality in bacterial communities within Shupaisha mangrove sediment, consistent with previous researches (Ma et al., 2020; Quintero et al., 2022; Ho et al., 2024). However, despite higher alpha diversity indices in autumn compared to spring, bacterial community structures showed the relatively high similarity between the two seasons (Figure 3A). O’Sullivan et al. reported minimal differences in geochemical factors between two different seasonal time points with an average temperature difference of 8°C in an estuary, suggesting a weak seasonal influence on the geochemical profiles of coastal wetland sediment (O’Sullivan et al., 2013). Similarly, the observed similarity in bacterial communities between spring and autumn could be also attributed to minor geochemical differences (Zhou et al., 2017). Furthermore, both Venn diagram with higher abundances of special OTUs in summer and winter (Figure 3D) and PCA analysis, based on weighted UniFrac and Bray-Curtis distance matrix method (Figure 3A), revealed the distinct grouping of summer and winter samples. Therefore, the four seasons exhibited a transitional pattern in bacterial community dynamics, suggesting except for pollution status, seasonal fluctuations in environmental factors can significantly influence microbial diversity and community component (Zhou et al., 2017; Bag et al., 2022; Fernandes et al., 2022).

Unlike previous studies, Actinobacteria were the second most abundant phylum in Shupaisha mangrove sediment, particularly during winter (13.27%) and spring (14.36%) (Figure 2A). Meanwhile, the relative abundance of Actinobacteria showed a significant negative correlation with temperature and pH (Figure 5A). Actinobacteria thrive in neutral or slightly alkaline soils and efficiently degrade organic matter such as cellulose, hemicellulose, pectin, and chitin (Law et al., 2019). They are also prolific producers of secondary metabolites with various bioactivities, including antimicrobial, anticancer, antioxidant, and immunosuppressive activities (Law et al., 2020; Ye et al., 2023). While not essential for bacterial growth, the production of secondary metabolites contributes to host defense and survival under unfavorable environmental conditions (Law et al., 2019). Although there were no significant seasonal differences in sediment particle size, TC and TN, lower temperature and pH and potentially unidentified factors likely created less favorable conditions for other bacterial groups in winter and spring, leading to the higher proportion of Actinobacteria.

Bacteroidetes was significantly more abundant in summer than in other seasons. A highly significant positive correlation (*p* < 0.01) between Bacteroidetes and temperature was observed (Figure 5), indicating increased relative abundance with rising summer temperatures. High temperature and rainfall fluctuations in summer likely contributed to fluctuating soil moisture. Elevated moisture content is known to favor Bacteroidetes growth, explaining the observed seasonal dominance (Klotz and Stein, 2008; Stein et al., 2013). In contrast, Firmicutes abundance negatively correlated with temperature (*p* < 0.05, Figure 5), showing a seasonal pattern: winter (2.26%) > autumn (2.05%) > spring (1.79%) > summer (0.96%). This preference for cooler temperatures is consistent with Firmicutes thriving in sub-zero conditions (Hultman et al., 2015). However, the study area’s year-round average temperature of approximately 18°C explains their relatively low abundance and lack of dominance. pH and salinity are also important environmental factors influencing mangrove bacterial community structure. While previous studies have documented their impact on soil and sediment bacterial communities (Hollister et al., 2010; Estrelles et al., 2015; Liu et al., 2015), this study found a significant pH correlation only with Actinobacteria, although correlations with other groups were observed but not significant (Figure 5). This aligns with previous finding that pH effect on bacterial community composition may be masked by other environmental factors acting in concert (Zhang et al., 2016). Furthermore, high salinity can cause osmotic stress, leading to water loss and bacterial mortality in sensitive bacteria, while low salinity can cause cell lysis due to excessive water uptake (Estrelles et al., 2015). However, this study revealed no significant salinity effect on dominant bacterial phyla, and only Gemmatimonadetes and Lentisphaerae showed significant positive correlations (*p* < 0.05) with salinity, while Nitrospirae exhibited a highly significant negative correlation (*p* < 0.05, Figure 5).

## 5 Conclusion

This study revealed significant seasonal patterns in the structure and diversity of microbial community in Shupaisha mangrove. Winter and summer samples exhibited significantly higher richness and Shannon indices than spring and autumn samples. Bacterial communities differed substantially between winter and summer, but showed high similarity between spring and autumn, suggesting a transitional pattern in bacterial community dynamics. Furthermore, Proteobacteria (23.59–44.40%), Actinobacteria (4.92–19.01%), and Bacteroidetes (4.31–22.79%) were identified as dominant phyla. Actinobacteria were more abundant during winter and spring, while Bacteroidetes was highest in summer, exhibiting a significant positive correlation with temperature. Multiple environmental factors, including temperature, sand content, and pH, greatly influencing mangrove bacterial community structure and likely mediated the seasonal patterns. These findings contribute to a deeper understanding of the diversity and composition patterns of sediment microbial communities in the Shupaisha mangrove, providing valuable insights for the ecological health and sustainable development of the Shupaisha Island.

## Data Availability

The datasets presented in this study can be found in online repositories. The names of the repository/repositories and accession number(s) can be found in the article/Supplementary material.
