# Supplementary material for: Seasonal dynamics of bacterial communities in mangrove sediments of Shupaisha island, Zhejiang Province, China
Source: Front Microbiol. 2025 Feb 19;16:1526730. doi: 10.3389/fmicb.2025.1526730 (PMC11880258; doi:10.3389/fmicb.2025.1526730)
Supplement: Supplementary file 1 [file Data_Sheet_1.docx]

Supplementary Material

# Supplementary Data

All the original sequences used in the article have been uploaded to the NCBI official website with the serial number PRJNA1133986.

# Supplementary Figures


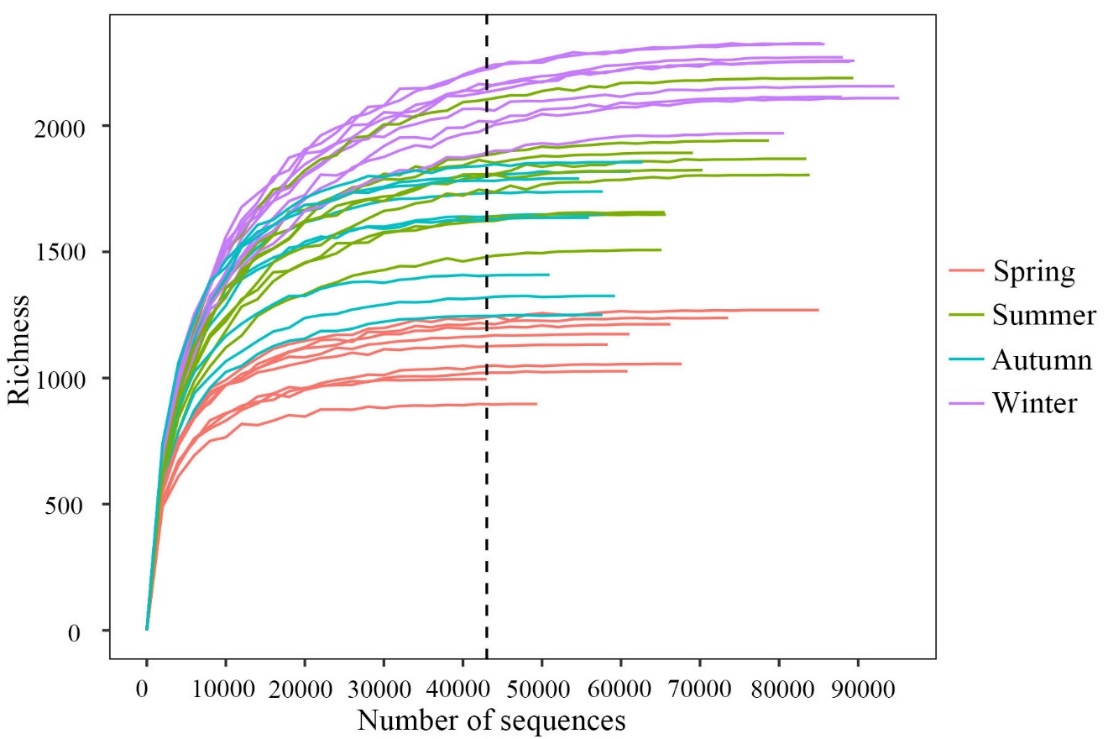


Supplementary Figure 1. Dilution curves of bacterial communities in sediment samples (Richness)


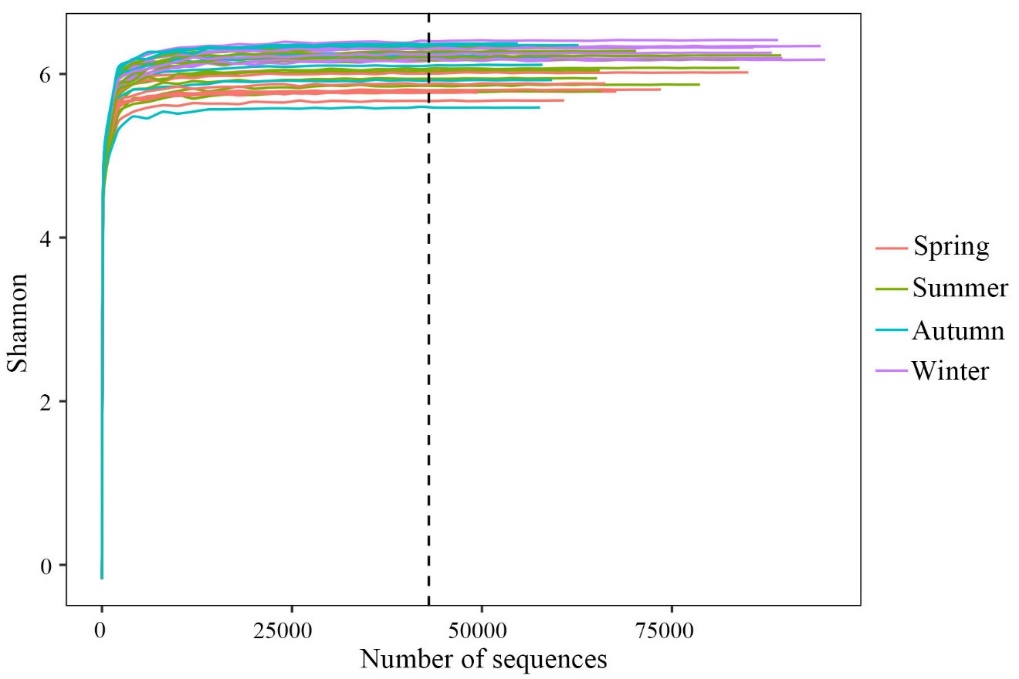


Supplementary Figure 2. Dilution curves of bacterial communities in sediment samples (Shannon)


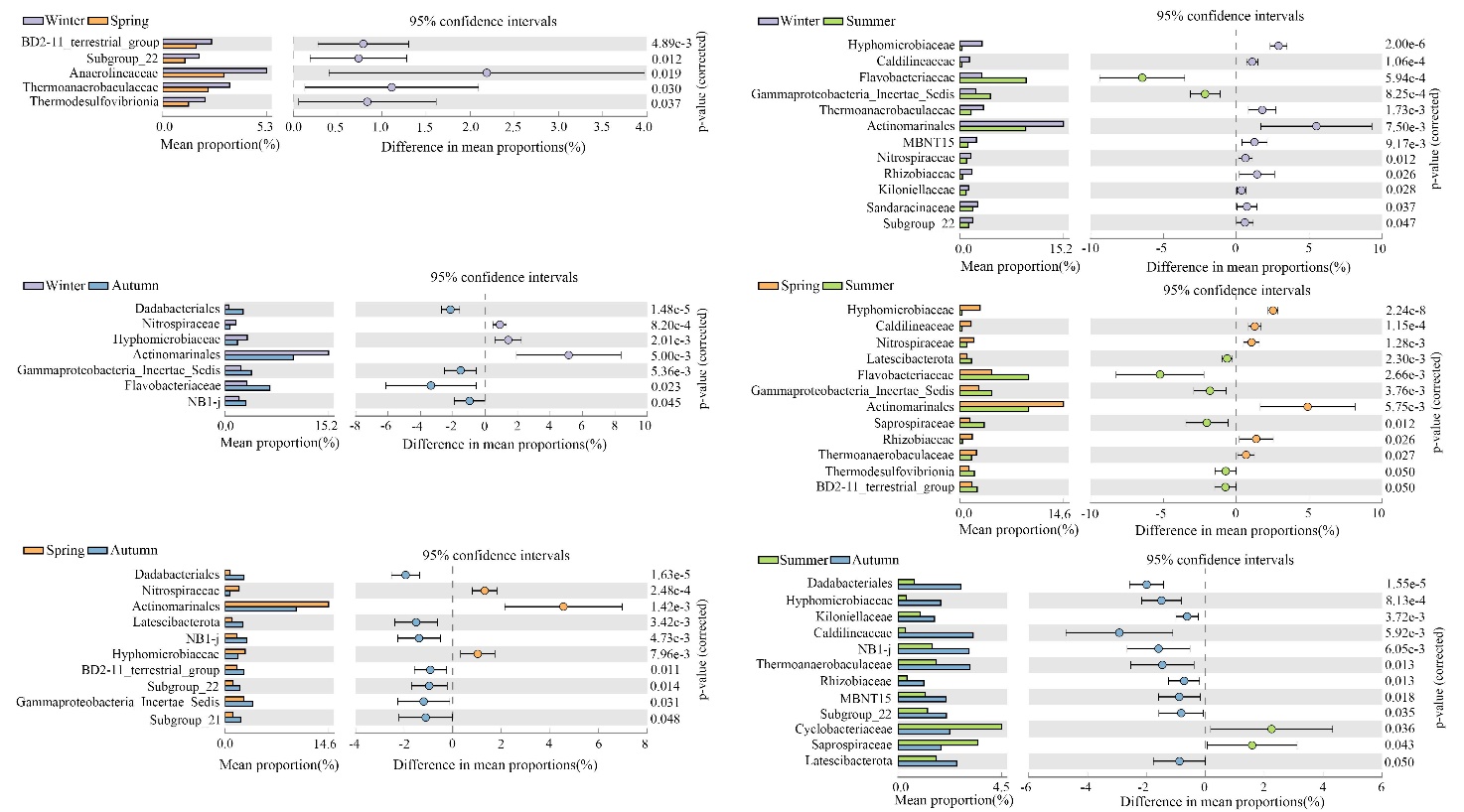


Supplementary Figure 3. Bacteria families with significant differences in relative abundance between seasons


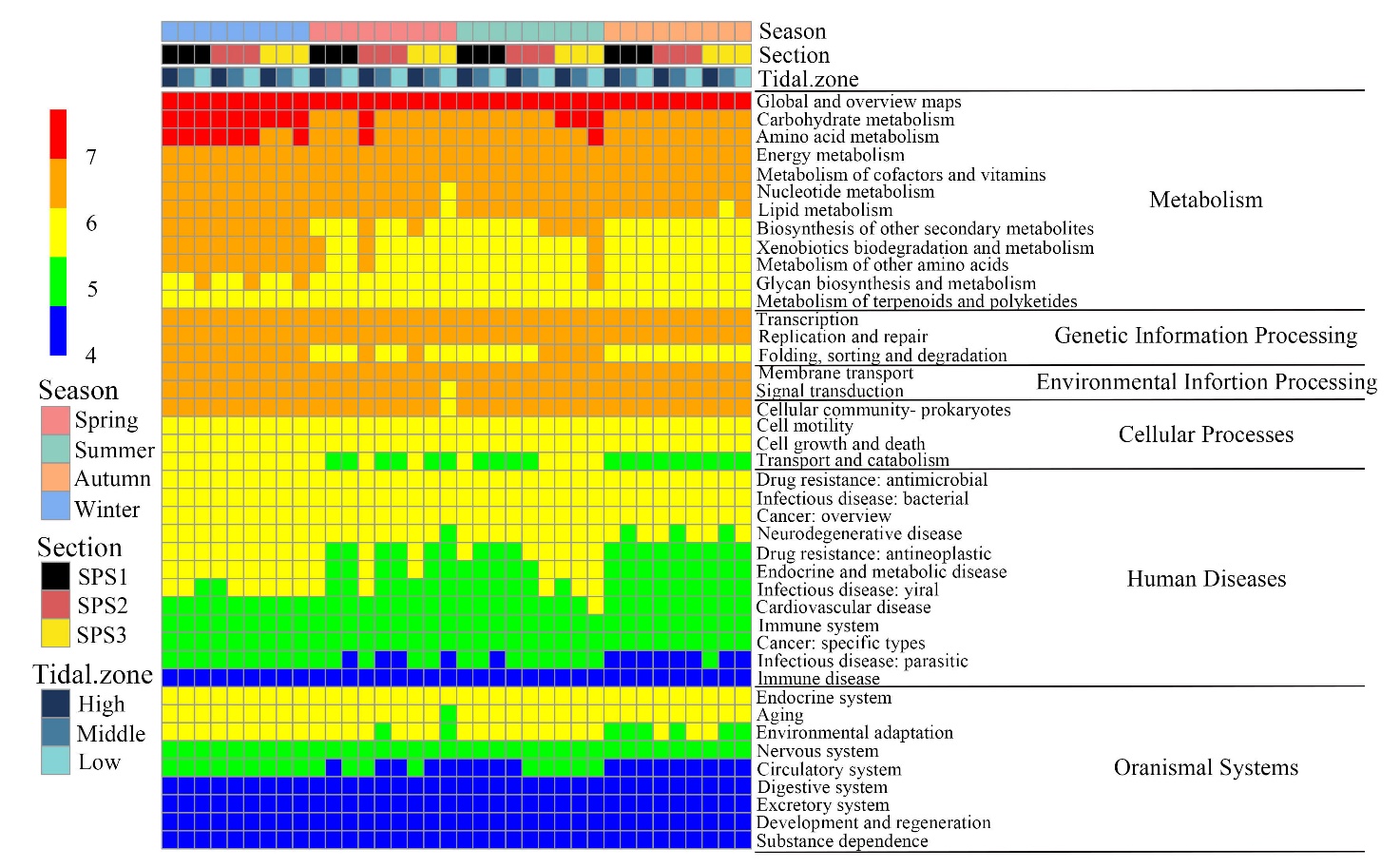


Supplementary Figure 4. Heatmap showing the distributions of bacterial functions that were assigned by PICRUSt2 across season

# Supplementary Tables

Supplementary Table 1. Bacterial community diversity index of mangrove sediments in this study

| Season | Sample | Richness | Shannon | Simpson | Pielou | goods_coverage |
| --- | --- | --- | --- | --- | --- | --- |
| Winter | S1H2111 | 2114 | 9.138 | 0.996 | 0.827 | 1 |
|  | S1M2111 | 2323 | 9.121 | 0.994 | 0.816 | 1 |
|  | S1L2111 | 2156 | 9.148 | 0.994 | 0.826 | 1 |
|  | S2H2111 | 2254 | 9.254 | 0.995 | 0.831 | 1 |
|  | S2M2111 | 2326 | 9.130 | 0.995 | 0.816 | 1 |
|  | S2L2111 | 2109 | 8.906 | 0.993 | 0.807 | 1 |
|  | S3H2111 | 1970 | 8.926 | 0.994 | 0.816 | 1 |
|  | S3M2111 | 2271 | 9.029 | 0.993 | 0.810 | 1 |
|  | S3L2111 | 2257 | 8.934 | 0.994 | 0.802 | 1 |
| Spring | S1H2205 | 1056 | 8.345 | 0.993 | 0.831 | 1 |
|  | S1M2205 | 1132 | 8.725 | 0.995 | 0.860 | 1 |
|  | S1L2205 | 1174 | 8.348 | 0.990 | 0.819 | 1 |
|  | S2H2205 | 1269 | 8.681 | 0.994 | 0.842 | 1 |
|  | S2M2205 | 897 | 8.327 | 0.994 | 0.849 | 1 |
|  | S2L2205 | 1027 | 8.188 | 0.988 | 0.818 | 1 |
|  | S3H2205 | 1238 | 8.380 | 0.989 | 0.816 | 1 |
|  | S3M2205 | 1213 | 8.485 | 0.992 | 0.828 | 1 |
|  | S3L2205 | 995 | 8.333 | 0.993 | 0.837 | 1 |
| Summer | S1H2208 | 1507 | 8.580 | 0.993 | 0.813 | 1 |
|  | S1M2208 | 1657 | 8.717 | 0.993 | 0.815 | 1 |
|  | S1L2208 | 1648 | 8.352 | 0.988 | 0.782 | 1 |
|  | S2H2208 | 1892 | 9.125 | 0.995 | 0.838 | 1 |
|  | S2M2208 | 1824 | 9.060 | 0.995 | 0.836 | 1 |
|  | S2L2208 | 1941 | 8.469 | 0.987 | 0.775 | 1 |
|  | S3H2208 | 1869 | 8.923 | 0.994 | 0.821 | 1 |
|  | S3M2208 | 1805 | 8.763 | 0.993 | 0.810 | 1 |
|  | S3L2208 | 2189 | 8.983 | 0.992 | 0.810 | 1 |
| Autumn | S1H2211 | 1739 | 9.034 | 0.993 | 0.839 | 1 |
|  | S1M2211 | 1635 | 9.153 | 0.996 | 0.857 | 1 |
|  | S1L2211 | 1649 | 8.816 | 0.991 | 0.825 | 1 |
|  | S2H2211 | 1818 | 8.956 | 0.992 | 0.827 | 1 |
|  | S2M2211 | 1409 | 8.973 | 0.995 | 0.858 | 1 |
|  | S2L2211 | 1250 | 8.065 | 0.984 | 0.784 | 1 |
|  | S3H2211 | 1325 | 8.548 | 0.993 | 0.824 | 1 |
|  | S3M2211 | 1790 | 9.193 | 0.994 | 0.851 | 1 |
|  | S3L2211 | 1855 | 9.163 | 0.994 | 0.844 | 1 |

Supplementary Table 2. Sedimentary environmental parameters of different seasons at each sampling site in this study

| Sample | Date | Longitude (E) | Latitude (N) | Temperature (℃) | Salinity (ppt) | pH | Clay (%) | Silt (%) | Sand (%) | TN (mg/g) | TC (mg/g) | TC/TN |
| --- | --- | --- | --- | --- | --- | --- | --- | --- | --- | --- | --- | --- |
|  |  |  |  |  |  |  |  |  |  |  |  |  |
| S1H2111 | 2021.12.04 | 120°51'58.92" | 27°56'55.17" | 13.90 | 18.95 | 7.78 | 34.23 | 59.72 | 6.04 | 1.265 | 12.621 | 9.976 |
| S1M2111 | 2021.12.04 | 120°52'0.33" | 27°56'56.14" | 14.50 | 20.97 | 7.82 | 33.20 | 57.76 | 9.03 | 1.217 | 13.419 | 11.025 |
| S1L2111 | 2021.12.04 | 120°52'1.42" | 27°56'56.68" | 14.40 | 18.16 | 7.86 | 35.76 | 62.92 | 1.31 | 1.014 | 11.956 | 11.789 |
| S2H2111 | 2021.12.04 | 120°51'42.09" | 27°57'4.84" | 14.50 | 18.63 | 7.75 | 33.94 | 58.77 | 7.28 | 1.075 | 12.191 | 11.344 |
| S2M2111 | 2021.12.04 | 120°51'43.23" | 27°57'6.15" | 13.50 | 20.80 | 7.88 | 37.83 | 62.17 | 0.00 | 1.317 | 13.481 | 10.234 |
| S2L2111 | 2021.12.04 | 120°51'46.03" | 27°57'8.93" | 14.30 | 17.05 | 7.98 | 32.07 | 67.74 | 0.18 | 0.983 | 11.187 | 11.381 |
| S3H2111 | 2021.12.04 | 120°51'26.54" | 27°57'15.06" | 13.30 | 22.05 | 7.69 | 37.86 | 60.92 | 1.21 | 1.085 | 11.404 | 10.507 |
| S3M2111 | 2021.12.04 | 120°51'27.00" | 27°57'16.92" | 13.20 | 20.73 | 7.97 | 37.17 | 62.36 | 0.46 | 1.274 | 15.037 | 11.804 |
| S3L2111 | 2021.12.04 | 120°51'28.61" | 27°57'18.61" | 13.80 | 25.67 | 7.37 | 34.48 | 64.75 | 0.76 | 1.204 | 13.494 | 11.210 |
| S1H2205 | 2022.05.15 | 120°51'59.14" | 27°56'55.06" | 18.90 | 12.08 | 7.17 | 39.72 | 58.94 | 1.33 | 1.250 | 14.324 | 11.464 |
| S1M2205 | 2022.05.15 | 120°52'0.31" | 27°56'56.04" | 18.80 | 13.16 | 7.05 | 40.60 | 59.21 | 0.98 | 1.213 | 14.003 | 11.548 |
| S1L2205 | 2022.05.15 | 120°52'2.05" | 27°56'56.43" | 18.30 | 11.97 | 7.33 | 39.64 | 59.74 | 0.61 | 1.095 | 12.205 | 11.151 |
| S2H2205 | 2022.05.15 | 120°51'41.81" | 27°57'4.87" | 19.30 | 12.86 | 7.05 | 40.81 | 58.38 | 0.80 | 1.171 | 13.008 | 11.108 |
| S2M2205 | 2022.05.15 | 120°51'43.10" | 27°57'5.86" | 19.60 | 12.73 | 7.17 | 40.18 | 59.22 | 0.59 | 1.144 | 12.675 | 11.080 |
| S2L2205 | 2022.05.15 | 120°51'45.63" | 27°57'9.26" | 18.10 | 10.84 | 7.46 | 39.66 | 58.30 | 2.03 | 1.037 | 12.194 | 11.758 |
| S3H2205 | 2022.05.15 | 120°51'25.06" | 27°57'16.29" | 17.80 | 11.29 | 7.59 | 36.35 | 63.21 | 0.43 | 1.284 | 13.481 | 10.498 |
| S3M2205 | 2022.05.15 | 120°51'25.77" | 27°57'17.06" | 17.20 | 11.24 | 7.64 | 38.50 | 60.94 | 0.55 | 1.269 | 13.492 | 10.636 |
| S3L2205 | 2022.05.15 | 120°51'27.29" | 27°57'19.39" | 18.50 | 18.73 | 6.85 | 41.51 | 57.11 | 1.37 | 1.049 | 11.626 | 11.081 |
| S1H2208 | 2022.08 | 120°51'58.98" | 27°56'54.63" | 29.80 | 14.50 | 7.63 | 43.33 | 56.67 | 0.00 | 1.300 | 14.265 | 10.972 |
| S1M2208 | 2022.08 | 120°52'1.64" | 27°56'57.03" | 30.00 | 25.30 | 7.91 | 32.36 | 67.27 | 0.37 | 0.910 | 12.718 | 13.973 |
| S1L2208 | 2022.08 | 120°52'2.05" | 27°56'56.43" | 30.60 | 21.10 | 7.32 | 35.44 | 64.45 | 0.10 | 1.035 | 11.739 | 11.337 |
| S2H2208 | 2022.08 | 120°51'46.54" | 27°57'2.28" | 31.40 | 23.90 | 8.66 | 41.64 | 57.96 | 0.39 | 1.387 | 14.272 | 10.290 |
| S2M2208 | 2022.08 | 120°51'45.84" | 27°57'4.53" | 30.02 | 22.30 | 8.78 | 38.98 | 60.14 | 0.87 | 1.213 | 14.695 | 12.119 |
| S2L2208 | 2022.08 | 120°51'48.46" | 27°57'7.66" | 29.20 | 19.00 | 7.81 | 37.38 | 61.27 | 1.34 | 1.015 | 11.284 | 11.118 |
| S3H2208 | 2022.08 | 120°51'25.34" | 27°57'15.21" | 39.30 | 22.50 | 8.26 | 40.30 | 58.70 | 1.00 | 1.156 | 12.406 | 10.734 |
| S3M2208 | 2022.08 | 120°51'25.91" | 27°57'16.35" | 31.02 | 21.90 | 7.69 | 39.11 | 58.66 | 2.23 | 1.091 | 11.440 | 10.482 |
| S3L2208 | 2022.08 | 120°51'27.26" | 27°57'18.35" | 30.10 | 19.70 | 7.62 | 39.31 | 60.09 | 0.59 | 1.061 | 11.829 | 11.145 |
| S1H2211 | 2022.10.30 | 120°51'59.14" | 27°56'55.06" | 19.10 | 33.80 | 7.53 | 35.20 | 64.80 | 0.00 | 1.171 | 11.911 | 10.168 |
| S1M2211 | 2022.10.30 | 120°52'0.31" | 27°56'56.04" | 19.80 | 32.00 | 7.44 | 39.95 | 56.77 | 3.28 | 1.002 | 11.305 | 11.282 |
| S1L2211 | 2022.10.30 | 120°52'15.57" | 27°56'46.56" | 19.90 | 26.60 | 8.08 | 39.45 | 59.36 | 1.18 | 1.027 | 12.286 | 11.962 |
| S2H2211 | 2022.10.30 | 120°51'41.81" | 27°57'4.87" | 19.50 | 32.50 | 7.28 | 36.66 | 63.34 | 0.00 | 1.186 | 12.377 | 10.433 |
| S2M2211 | 2022.10.30 | 120°51'43.10" | 27°57'5.86" | 20.60 | 30.30 | 7.24 | 40.90 | 59.10 | 0.00 | 1.093 | 11.560 | 10.580 |
| S2L2211 | 2022.10.30 | 120°52'3.16" | 27°56'54.90" | 20.10 | 26.70 | 8.08 | 42.80 | 57.20 | 0.00 | 1.012 | 12.205 | 12.062 |
| S3H2211 | 2022.10.30 | 120°51'25.06" | 27°57'16.29" | 19.80 | 35.60 | 7.33 | 35.26 | 64.46 | 0.28 | 1.756 | 21.749 | 12.386 |
| S3M2211 | 2022.10.30 | 120°51'25.77" | 27°57'17.06" | 20.00 | 33.30 | 7.30 | 41.32 | 58.18 | 0.50 | 1.012 | 11.373 | 11.235 |
| S3L2211 | 2022.10.30 | 120°51'27.29" | 27°57'19.39" | 20.80 | 23.40 | 7.95 | 37.51 | 61.30 | 1.18 | 0.980 | 11.171 | 11.405 |
